# Daily and seasonal heat usage patterns analysis in heat networks

**DOI:** 10.1038/s41598-022-13030-6

**Published:** 2022-06-02

**Authors:** L. Minh Dang, Sujin Lee, Yanfen Li, Chanmi Oh, Tan N. Nguyen, Hyoung-Kyu Song, Hyeonjoon Moon

**Affiliations:** 1 Department of Information and Communication Engineering, and Convergence Engineering for Intelligent Drone, Sejong University, Seoul, Republic of Korea; 2 Department of Artificial Intelligence, Sejong University, Seoul, Republic of Korea; 3 Department of Computer Science and Engineering, Sejong University, Seoul, Republic of Korea; 4 Department of Architectural Engineering, Sejong University, 209 Neungdong-ro, Gwangjin-gu, Seoul, 05006 Republic of Korea

**Keywords:** Energy harvesting, Energy infrastructure, Energy storage

## Abstract

Heat usage patterns, which are greatly affected by the users' behaviors, network performances, and control logic, are a crucial indicator of the effective and efficient management of district heating networks. The variations in the heat load can be daily or seasonal. The daily variations are primarily influenced by the customers' social behaviors, whereas the seasonal variations are mainly caused by the large temperature differences between the seasons over the year. Irregular heat load patterns can significantly raise costs due to pricey peak fuels and increased peak heat load capacities. The in-depth analyses of heat load profiles are regrettably quite rare and small-scale up until now. Therefore, this study offers a comprehensive investigation of a district heating network operation in order to exploit the major features of the heat usage patterns and discover the big factors that affect the heat load patterns. In addition, this study also provides detailed explanations of the features that can be considered the main drivers of the users' heat load demand. Finally, two primary daily heat usage patterns are extracted, which are exploited to efficiently train the prediction model.

## Introduction

A district heating (DH) network is a vital energy infrastructure that has been introduced and modernized during the last few decades as an efficient means to deliver heat to customers. Various types of strategic heat sources can currently be used in the DH networks, such as waste-to-energy plants^1^, combined heat and power (CHP) systems^2^, solar thermal energy (STE)^3^, heat pumps^4^, and geothermal power plants^5^. DH networks have many advantages compared to the other space heating options, such as the high heat production rate, low carbon footprint, possibility of integrating various heat sources, and customer-centric. DH systems can be applied to multiple application domains, ranging from small-scale systems for isolated communities or remote villages to massive systems that supply heat to major cities^6^. Therefore, they play an essential part in the future smart energy grids.

Heat usage in DH networks include heat usage from the consumers and the distribution losses^7^. The engineers of DH plants try to offer a stable heat supply, but the consumers’ heat usage patterns are not constant. Therefore, variations in heat usage at the customer’s end can lead to heat load variations in the heating system. In order to obtain more in-depth knowledge in regards to DH networks, please refer to^8^. Another crucial factor that significantly affects the heat load is seasonal usage. A distinctive outdoor temperature between winter and summer creates significant heat usage variations over time. In addition, daily customer heat usage behavior also causes heat load variations^7^.

As a result, the current reactive management-based DH networks must be converted into model predictive-based management. Heat usage patterns prediction is the biggest challenge in order to facilitate an effective model predictive-based DH network. It becomes possible to optimize the overall heat production, lower grid losses, and enhance the energy usage efficiency with an accurate heat usage patterns prediction model^9^. Another practical factor that raises the requirement for heat usage forecasting is that the heat supplied to the customers must match their real-time demand in order to ensure the distribution temperature is in an acceptable range^10^. Therefore, it is imperative to develop an advanced heat usage patterns prediction model.

The previous heat usage patterns prediction can be grouped into two main groups, including physical energy (i.e., white box) and data-driven approaches (i.e., black box). The physical energy approach relies entirely on analyzing functional correlations of building parameters to build the heat load profile^11^. Although the physical energy-based models' forecast accuracy is usually better than the data-driven models, it is labor-intensive and time-consuming to develop correct physical energy profiles for each building. On the other hand, data-based approaches construct prediction models by learning the underlying relations of heat usage and other influential factors based on the DH historical data. Due to the rapid development of big data technologies such as smart metering has obtained a growing interest in recent years. By taking full advantage of the advancement of artificial intelligence (AI) in recent years^12,13^, the data-based heat usage prediction models show promising results^7^. Moreover, deep learning, which is a specialized area of Machine Learning (ML) that allow computers to learn from and make predictions about data automatically, has progressively been a default choice in various domains, such as Computer Vision (CV)^14^ and natural language processing (NLP)^15^. Commonly used ML algorithms in heat usage patterns predictions include moving average^16^, multiple linear regression^17^, regression tree^18^, support vector regression (SVR)^19^, extreme gradient boosting (XGBoost)^20^, and deep learning^15^. However, the data-driven approach has some weaknesses, such as poor performance on unknown datasets due to the variety of DH networks and historical data. In addition, most data-driven models are called the "black-box" model because they cannot explain why a specific output is reached^21^.

The heat usage patterns of the network are a pressing problem that needs to be solved in order to facilitate a precise and efficient DH operation and control^22^. Previously, the rarity of high-resolution, hourly, or sub-hourly meter data before installing smart meters led to a limited number of studies concentrating on the analysis of heat usage patterns in DH systems. For instance, the heat usage patterns were analyzed in order to calculate heat usage capacities for billing objectives in^17,23^. Energy consumption was predicted in^24^ to increase energy efficiency in residential householders. The energy signature (ES) approach has been studied extensively for representing heat usage patterns of a single building in numerous research for various objectives, such as temperature-based^25^, heat loss estimation^26^, and abnormality detection^27^. However, it relies solely on outdoor temperature to reflect the heat usage pattern of individual buildings yearly. It also fails to work with other parameters, such as daily behavior and weekend routines. Some studies have recently concentrated on peak prediction^28^ with the primary goal of conserving energy through reducing daily peak patterns of the heat load curves. However, the peak of heat usage depends mainly on weather conditions and may reflect the momentary behaviors of customers.

The important influencers of heat usage can primarily be grouped into three categories, which include meteorological factors, time factors, and clients' social behaviors^8^. Meteorological factors contain influential information, such as outdoor temperatures, humidity, and wind speeds. Time factors refer to various variables, such as the year, month, day, and hour. Historical heat usage data can also be an important factor in regard to correlating the historical and future heat loads^7^. Finally, the clients' social behaviors, which are closely related to the time factors, can also cause heat usage variations in terms of daily or seasonal. These factors hugely affect the heat usage patterns, but the previous studies did not sufficiently apply them.

This study is proposed to address issues that have been partially addressed in previous studies, such as heat load prediction and heat usage analysis. In addition, it also investigates unsolved issues, such as data-based pattern analysis, daily heat variation analysis, and crucial factors affecting heat usage. The main contributions include (a) categorizing heat usage patterns into daily and seasonal patterns and analyzing them separately, (b) extracting representative patterns showing the characteristics of distinctive daily heat load variation based on the clustering technique, and (c) providing some explanations for data-driven heat usage prediction model, which was ignored in previous work.

The paper is organized as follows. “Dataset” Section describes the dataset and various preprocessing techniques implemented in this study. Next, “Methodology” section describes the proposed framework. After that, various experiments are conducted to thoroughly illustrate the effectiveness of the proposed framework in “Experimental results” section. Finally, “Conclusion” section summarizes the research and mentions the future directions.

## Dataset

The main dataset used in this study was gathered from an eco-friendly gas-fired power combined heat power and cooling (CHP) plant in Chuncheon, Korea, that supplies electricity and high-efficiency local heating and cooling. The CHP plant adds approximately 470 MW to the grid and 120 Gigacalories (Gcal) of local cooling and heating for 24,000 households. CHP is an efficient and cleaner approach to producing power and thermal energy from a single fuel source. By applying state-of-the-art denitrification and hybrid cooling systems to the Chuncheon CHP, environmental pollution was reduced to 1/3 of the emission standard.

The dataset contains the hourly average heat load between January 2014 to November 2018, which spans 4 heating seasons. The heating season usually indicates the period from November 1st to April 15th^8^. The heat load indicates the energy amount transmitted by the grid to the clients at a fixed time. It is not related to the physical energy flow but rather the total energy provided to various network locations, involving both space heating (SH) and domestic hot water (DHW). The heat load data from the dataset also includes network losses. The heat usage is constantly estimated hourly. Therefore, there are 8760 hourly heat load values per year. The heat load unit is Gcal, a common unit of measurement of heat energy in heating systems^29^. 1 Gcal is equal to 1,163 kilowatt-hours (kWh). The yearly heat usage data is utilized in order to investigate the daily heat usage patterns and produce some explanations for the model's outputs in the experimental result section.

Corresponding hourly weather data for Chuncheon were also obtained in order to analyze the ES as additional measures for the heat load dataset to explore the potential relationships with the heat load data. The available data includes the outdoor temperatures, average wind speeds, and humidity.

The dataset contains approximately 43,080 entries, which include dates, temperatures, humidity, and wind speeds as the input features and the average heat usage as the output feature. Sometimes, a single value or a series of fewer than 5 values in a row is missing from the average heat usage data. Those missing values are reconstructed using interpolation, because the DH networks are thermally stable with slow heat changes. On the other hand, missing weather data values, such as temperature and humidity, are replaced by the corresponding data from either the day before or the day after the date under consideration, because no significant changes in the daily weather data exist. The portion of the filled data is less than one per cent, which does not significantly influence the final results.

## Methodology

**Figure 1:**
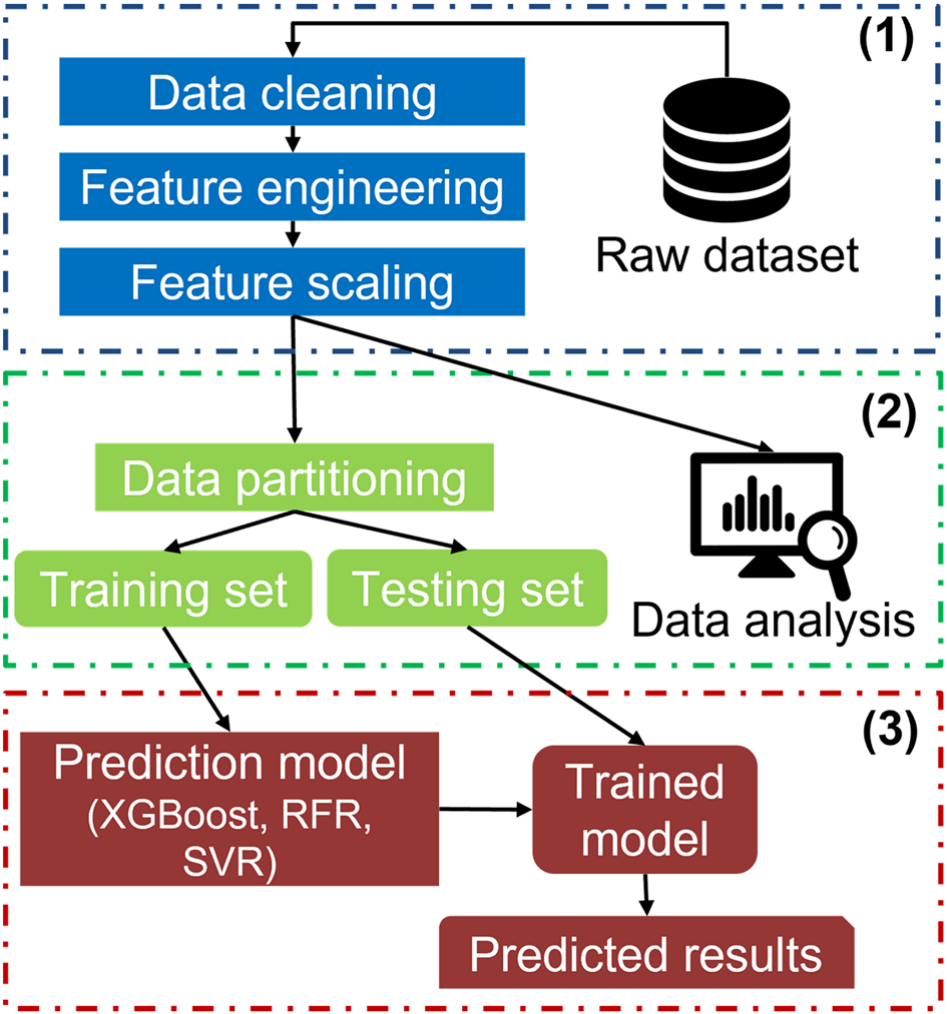
The overall architecture of the heat load analysis framework.

Figure 1 illustrates the three main stages of the heat load analysis system, which include (1) data pre-processing, (2) data analysis and partitioning, and (3) heat load pattern forecasting.

- Data pre-processing phase: The collected raw heat usage data host various issues, such as missing values, null values, and duplicate data, which can affect the training process. As a result, it is imperative to perform pre-processing in order to transform the data into a normalized and standard format.
- Data analysis and partitioning phases: The data analysis is crucial in understanding the datasets especially for the heat usage analysis topic, but the previous studies did not seriously consider the analysis of the datasets. As a result, various data analysis methods are implemented in the study to learn about the data before the training process. After that, the data is split into the training and testing datasets.
- Heat load forecasting: In this section, various ML algorithms, such as SVR, MLP, and Boosting, are deployed to perform the heat usage forecasting. Hourly heat usage information and meteorological data are extracted as the input features, which is useful in regards to improving the learning process of the complex features that use those algorithms.

### Data pre-processing

#### Data cleaning

Data transmission losses are inevitable, so the collected data usually includes some empty values, which significantly reduce the dataset's quality if they are not properly handled. Moreover, ML models fail to work if missing values exist in the dataset^21^. Therefore, data cleaning is implemented in order to process the missing entries in the heat load data, outdoor temperature, humidity, and wind speed in order to enhance the data quality. Various methods, such as imputation and simple Moving Average (MA) can be applied to fill the empty values.

First of all, the Exponential Weighted Moving Average (EWMA) method, which was developed based on the simple MA, is implemented in order to fill the missing heat load data from the surrounding values^16^. Unlike the simple moving average, EWMA places larger weighting on the most recent data points, whereas exponentially lower weight factors are placed on the older data points. The heat usage indicator fluctuates constantly, so the difference between the two near data points is considered small. After that, the missing weather data, such as wind speed, outdoor temperature, and humidity, are filled with the nearest value of the previous day, because the differences between two continuous dates are deemed minor.

As a result, this study applies EWMA to solve the heat usage empty values and the data from the previous date in order to fill the missing weather data.

#### Feature engineering

Feature engineering is a required process that is applied to the data when an input feature is a categorical feature. Most ML methods favor numerical data, so it is better to convert all the categorical data into numerical data. One-hot encoding, which produces a binary representation for every category of a categorical column, is a standard approach that is applied to treat the categorical features^30^.

**Figure 2:**
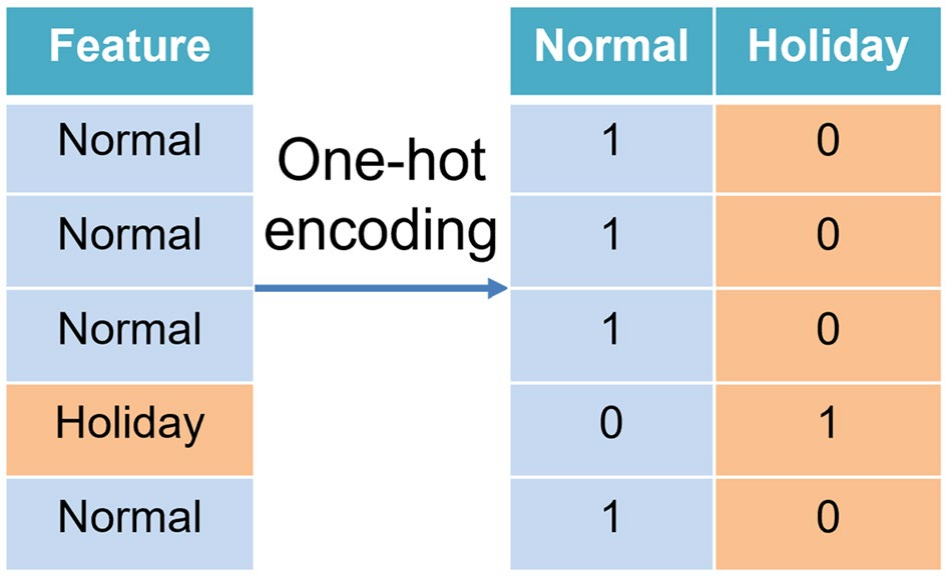
A sample one-hot encoding approach on the holiday feature.

Using the holiday feature from the proposed dataset as an example, two distinctive values indicate whether the day under consideration is *normal* or *holiday*, as displayed in Fig. 2. The original holiday categorical feature is divided into two binary features, which include *normal* and *holiday*, with one-hot coding implemented. When the feature value of the holiday feature of a data instance is *normal*, then the corresponding value in the *normal* binary feature is assigned *1*, and the *holiday* binary feature is set as *0*. It suggests that the sample belongs to the *normal* category of the categorical feature. The contrary approach is applied to the case when the feature value of the holiday feature of a data instance is *holiday*.

#### Feature scaling

ML methods perform poorly with numerical input that has distinctive value ranges with occasional exceptions^31^. A well-known approach to this issue is feature scaling, which standardizes all the variables to a uniform scale. Standardization and min–max normalization are two typical feature scaling methods. The dataset that was used in this study contained abnormally high heat usage on some days (outliners), which has a big role in the heat usage analysis. As a result, this study used standardization for feature scaling, because min–max normalization reduces the effect of those outliners. The standardization method standardizes a feature by subtracting the mean and then scales it to unit variance.

### Data partitioning

Data partitioning is an imperative process that is used during the model’s development and evaluation. The original data is split into training and testing data. The training data is utilized for training and fine-tuning the prediction model, whereas the testing data is usually applied to investigate the model under various settings. Two types of heat usage variation, including daily and seasonal variations, will be investigated in this study.

Several distinctive daily heat usage patterns can be detected via cluster analysis. This section describes the cluster analysis for the daily heat usage data in order to identify unique users' heat usage patterns. After that, the dataset was divided into different training and testing sets using the extracted patterns.

#### Daily heat load variation

The demands from the clients of a DH system vary between the days, which lead to the daily heat usage variations. The geographical diversity and the fact that the heat usage for each customer reaches a peak at different times reduce the effect of the daily heat load variations. However, the heat load variations remain a crucial factor in DH systems, which is due to the social heat demands. The social heat demands can be created by individual or collective social behavior. For instance, whenever someone decides to use hot water to clean their hands, it contributes to a rise in the heat load of the building, which affects the DH network's heat resources.

Clustering is an unsupervised ML method that groups the data samples that have similar properties into different clusters^32^. Standard clustering approaches are hierarchical clustering and K-means. K-means clustering splits the samples into non-overlapping subsets, which are also referred to as clusters, whereas hierarchical clustering forms a set of nested clusters that are arranged as a tree without a predefined number of clusters. The clustering performance can be accessed using cluster validity indices, which include internal indices and external indices. Internal indices operate exclusively on the intrinsic information of the original clustering, whereas external indices use information about the ground truth^33^. Examples of internal indices are the Davies-Bouldin Index (DBI) and the Silhouette Index (SI), and examples of external indices are the Jaccard coefficient and the Rand index.

This research implements K-means clustering in order to recognize common patterns for daily heat usage. The performance of K-mean clustering is then evaluated based on the DBI, which is where a smaller DBI indicates a better clustering performance. K-means clustering is a matrix factorization problem, so the original heat usage data must be converted into a proper form before feeding it into the K-means clustering algorithm. The data is transformed into a $\mathrm{c}\times \mathrm{e}$ matrix. Where $\mathrm{c}$ represents each day, and $\mathrm{e}$ is the total heat usage amount used in $\mathrm{c}$. Each matrix's row indicates a daily heat usage profile, whereas each matrix's column denotes the heat load value recorded at a time step.

Stratified sampling is adopted in this study to split the original dataset into a training set (80%) and a testing set (20%) based on the output daily heat usage patterns.

#### Seasonal heat load variation

The seasonal heat load variation is transparent and easy to notice, which differs from the daily heat load variation. For instance, a contrastive outdoor temperature between summer and winter leads to the need to maintain a constant temperature inside the building envelope. In addition, people stay most of the time indoors in the winter and therefore require significantly more heat. On the contrary, people tend to spend more time outside during summer and holidays, so the heat usage decreases considerably during the summer and holidays.

The dataset contains the hourly average heat load between January 2014 to November 2018, which spans 4 heating seasons. The heating season usually indicates the period from November 1st to April 15th^8^. The data collected from 2014 to 2016 was used for training, and the period between 2017 to 2018 was utilized for evaluating the framework.

### Prediction model development

This section explains the SVR, XGBoost, and MLP models, which are utilized for training the heat usage prediction framework.

#### Support vector regression (SVR)

SVR is a standard regression algorithm used in statistical ML, which fits the training dataset well and correctly predicts the test data^34^. Unlike most common linear regression models, which have the main objective of minimizing the sum of the squared errors, SVR offers the flexibility to define how much error is acceptable and finds an optimal hyperplane to fit the data.

Heat usage prediction is a complicated non-linear problem due to several input features. As a result, a kernel function is applied to convert the complexed non-linear topic in the initial feature space into a linear topic in the high dimensional feature space, which is similar to the Support Vector Machines (SVM) algorithm. The SVR algorithm is then built on the converted feature space. The equation of the SVR algorithm on a multivariate set of N observations is described as follows.

1 $$\mathrm{y}=\sum_{n=1}^{N}({\alpha }_{n}-{\alpha }_{n}^{*})\mathrm{\varnothing }\left(\mathrm{x}\right)+\mathrm{b}$$

where ${\alpha }_{n}$ and ${\alpha }_{n}^{*}$ are nonnegative multipliers for each observation ${x}_{n}$ according to the dual formula; $\mathrm{\varnothing }$ indicate a kernel function, and $\mathrm{b}$ is a displacement of the dividing hyperplane.

In this research, the Gaussian Radial Basis function (RBF) was implemented as the kernel function, which is described using the equation supplied below.

2 $$K(xi,xj)=\mathrm{exp}\left(-\upgamma \Vert xi-xj\Vert 2\right)$$

where $\Vert xi-xj\Vert 2$ is the Euclidean distance between $xi$ and $xj$, and $\upgamma$ represents the Gamma parameter.

The training process of the SVR algorithm is considered to be a quadratic programming topic with the main goal of finding the coefficients that can be minimized.

3 $$L\left(\alpha \right)= \frac{1}{2}{\sum }_{i=1}^{N}{\sum }_{j=1}^{N}({\alpha }_{i}-{\alpha }_{i}^{*})({\alpha }_{j}-{\alpha }_{j}^{*})K\left(xi,xj\right)+\varepsilon {\sum }_{i=1}^{N}\left({\alpha }_{i}+{\alpha }_{i}^{*}\right)-{\sum }_{i=1}^{N}{y}_{i}({\alpha }_{i}-{\alpha }_{i}^{*})$$

subject to

4 $$\sum_{n=1}^{N}({\alpha }_{n}-{\alpha }_{n}^{*})=0$$

5 $$\forall \mathrm{n}:0\le {\alpha }_{n}\le \mathrm{C}$$

6 $$\forall \mathrm{n}:0\le {\alpha }_{n}^{*}\le \mathrm{C}$$

#### Extreme gradient boosting (XGBoost)

Boosting is an ensemble approach, which includes where additional classifiers are sequentially added in order to focus on incorrect samples that are predicted by the existing classifiers until no further improvements can be made^35^. Gradient boosting is proposed by creating new classifiers that analyze the residuals, which are considered the errors, of the previous models based on the gradient descent method that reduces the loss when adding new classifiers. Finally, the outputs from the created models are combined to output the final results.

XGBoost is a representative method for gradient tree boosting, which was proposed by Chen et al.^36^. Due to its speed and performance, it has recently become a default choice for structured or tabular data in applied ML fields and Kaggle competitions. XGBoost trains multiple Classification and Regression Trees (CART) and aggregates the predictions in order to create the final prediction, which is displayed in Eq. (1).

7 $$\widehat{{y}_{i}}={y}_{i}^{0}+\sigma \sum_{k=1}^{n}{f}_{k}({U}_{i})$$

where, $\widehat{{y}_{i}}$ represents the estimated prediction for the ${i}_{th}$ sample with ${U}_{i}$ as the parameter vector. ${y}_{i}^{0}$ indicates the computed mean of all the original parameters from the training samples. $\sigma$ controls the rate of adding additional trees to reduce over-fitting. $n$ is the number of estimators that correlate with independent trees for each ${f}_{k},$ which depicts the leaves weight that is established by minimizing the objective function $obj$ of the ${k}_{th}$ tree.

8 $$obj(th)=\sum_{i=1}^{n}L\left({y}_{i},{f}_{th}({x}_{i})\right)+\sum_{t=1}^{T}\Omega ({h}_{t})$$

where $\mathrm{L}$ represents the loss function that measures the difference between the ground truth ${y}_{i}$ and the predicted values ${f}_{th}({x}_{i})$. The root-mean-square error is implemented as the main loss function in this manuscript. The regularization parameter is used to smooth the learned weights and reduce the overfitting, which is defined by the equation provided below.

9 $$\Omega \left({h}_{t}\right)=rT+ \frac{1}{2}\lambda \sum_{j=1}^{T}{\mathrm{w}}_{j}^{2}$$

where $r$ and $\lambda$ are two regularization terms. T is the total number of leaves, and ${\mathrm{w}}_{j}^{2}$ is the score for each leaf.

#### Multilayer perceptrons (MLP)

MLP is a class of feedforward Artificial Neural Networks (ANN) that has a basic structure of one input layer, one or more hidden layers, and one output layer. An artificial neuron is the basic element of MLP, and every neuron in a current layer is connected to all neurons in the following layer. The number of layers and neurons are hyperparameters of MLP, and they need to be fine-tuned. MLP contains no recursive loop or feedback between the layers, which is unlike more complex deep learning structures, such as CNN and RNN. The data advances from the input layer to all the hidden layers and then to the output layer^37^. The general neuron-like processing unit process can be described using the following equation.

10 $$a=\mathrm{\varphi }\left(\sum_{j}{x}_{j}{w}_{j}+b\right)$$

where ${x}_{j}$ is the input data of the neuron, ${w}_{j}$ represents the learned weights, and $b$ indicates the bias. $\mathrm{\varphi }$ is a nonlinear activation function. The MLP computations can be expressed mathematically as follows.

11 $${H}^{(1)}={\mathrm{\varnothing }}^{(1)}\left(X{W}^{(1)\intercal }+1{b}^{(1)\intercal }\right)$$

12 $${H}^{(2)}={\mathrm{\varnothing }}^{(2)}\left({H}^{(1)}{W}^{(2)\intercal }+1{b}^{(2)\intercal }\right)$$

13 $$Y={\mathrm{\varnothing }}^{(3)}\left({H}^{(2)}{W}^{(3)\intercal }+1{b}^{(3)\intercal }\right)$$

where $X$ represents the entire input data. The matrix ${H}^{(ith)}$ contains the hidden units of the $ith$ layer for all the training data. ${\mathrm{\varnothing }}^{(ith)}$ indicates the activation function that is used in the $ith$ layer.

## Experimental results

### Hyperparameter fine-tuning

The three ML models described above have some hyperparameters, which must be defined before the training process. These hyperparameters are crucial, because they can improve the model performance if appropriately configured. This study implemented a grid search and fivefold cross-validation in order to fine-tune those parameters.

The required parameters for each algorithm are first defined, and the possible value ranges for each hyperparameter are then determined. After that, the grid search approach is conducted on all the possible combinations of the hyperparameters for each model, which figure out the best hyperparameter sets that help the model achieve the highest results^38^. Finally, fivefold cross-validation is performed to improve the framework robustness with the obtained optimal hyperparameters. It randomly splits the training data into 5 non-overlapping subsets of equal size. There are a total of 5 iterations, and 4 folds are utilized for training for each iteration, and the remaining fold is applied to perform the evaluation. The final output is the mean value of the 5 folds.

Table 1 illustrates the value ranges for each hyperparameter of each ML model under consideration and the optimized parameter value after performing the grid search approach.

**Table 1:** Predefined hyperparameter value ranges for each algorithm and the optimized values.

Model	Hyper parameters	Description	Considered values	Optimized values
XGBoost	$\sigma$	Learning rate	0.001, 0.005, 0.01, 0.1	0.1
	$n$	Number of estimators	50, 100, 200	100
	${d}_{tree}$	Max depth of $n$	1, 2, 3, …, 10	3
MLP	$\sigma$	Learning rate	0.001, 0.005, 0.01, 0.1	0.01
	${d}_{h}$	Number of hidden layers	2, 3, 4	2
	${M}_{j}$	Number of neurons in the hidden layer $j$	50, 100, 200	100
	${\mathrm{\varphi }}$	Activation function	ReLU, tanh	ReLU
SVR	$\mathrm{\varnothing }$	Regularization parameter	${10}^{0}, {10}^{1}, \dots , {10}^{5}$	${10}^{0}$
	$\Gamma$	Kernel coefficient	${10}^{-6}, {10}^{-5}, \dots , {10}^{-1}$	${10}^{-3}$

### Evaluation metrics

After the training process of the three different models for the heat usage prediction, three different evaluation metrics, which include the coefficient of determination (${R}^{2}$), mean absolute error (MAE), and root mean squared error (RMSE), are calculated to test the robustness of each model. The MAE depicts the average of the absolute difference between the ground truth and the prediction by measuring the average of the residuals. RMSE represents the square root of MSE, which estimates the standard deviation of the residuals. Finally, ${R}^{2}$ describes the proportion of the variance in the dependent variable that is estimated by the trained model. Lower MAE and RMSE values imply a better model performance. On the other hand, a higher ${R}^{2}$ value is considered better. Those metrics are described as follows.

14 $$MAE=\frac{1}{n}{\sum }_{i=1}^{N}\left|{y}_{i}-{\widehat{y}}_{i}\right|$$

15 $$RMSE=\sqrt{\frac{1}{n}{\sum }_{i=1}^{N}{\left({y}_{i}-{\widehat{y}}_{i}\right)}^{2}}$$

16 $${R}^{2}= 1-\frac{\sum {\left({y}_{i}-{\widehat{y}}_{i}\right)}^{2}}{\sum {\left({y}_{i}-\overline{y }\right)}^{2}}$$

where $N$ indicate the total number of instances in the testing set. ${y}_{i}$ is the ground truth and ${\widehat{y}}_{i}$ is the predicted value of the $i$ testing instance. Finally, $\overline{y }$ represents the mean value of all the ground truth data.

### Case study

#### Dataset partition that is based on the daily heat usage variation

K-means clustering is conducted to pinpoint the representative daily heat usage patterns in order to perform data partitioning for the daily heat usage analysis, which was previously explained. The hourly heat usage data is first converted into a matrix, which is where each row indicates the heat load profile for a day, and each column describes the hourly heat load profile. Next, the number of centroids $K$ from 2 to 10 are evaluated to find the optimal $K$ number.

**Figure 3:**
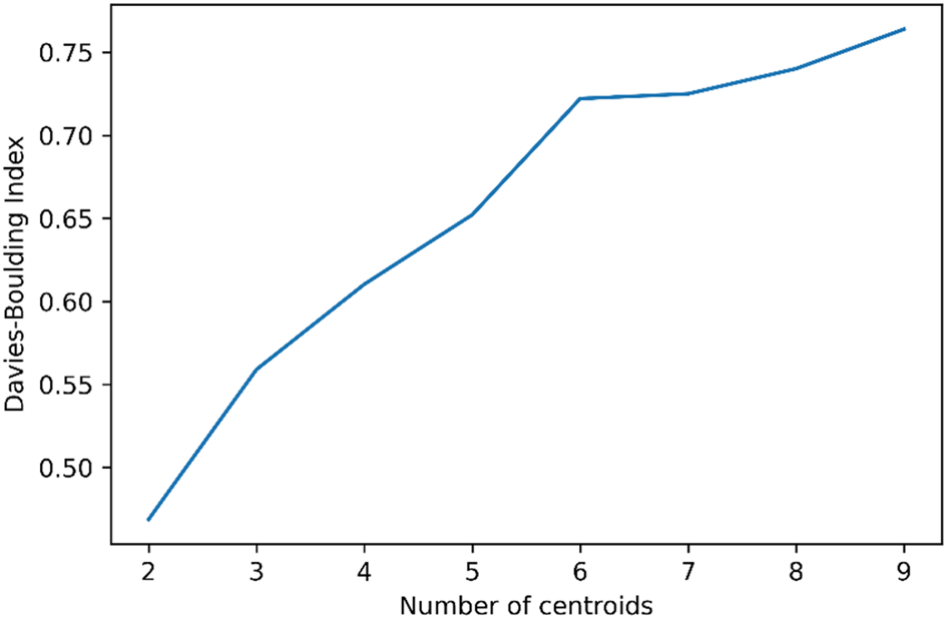
DBI graph for different number of centroids.

Figure 3 illustrates the DBI values for the different values of the K centroids. The smaller the DBI value is, the better the clustering performance is. The smallest DBI value, which is less than 0.15, is recorded when the number of centroids is set to 2. As a result, two distinctive daily heat usage patterns were extracted with the $K$ centroid value equal to 2. The first cluster, which is pattern 1, includes 1231-day samples, and the second cluster, which is pattern 2, has 563-day samples.

**Figure 4:**
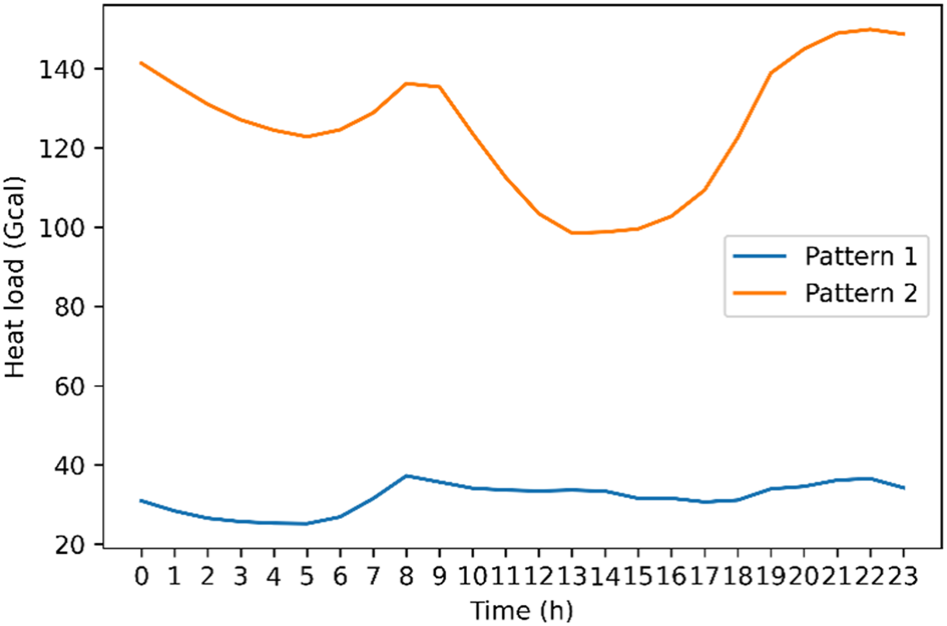
Notable patterns for the daily heat load variation using the clustering technique.

Figure 4 shows the average hourly heat usage for each pattern, which reveals a huge difference between the two identified heat usage patterns. Pattern 2 demonstrates an irregular heat usage pattern compared to the stable amount of heat that is being used throughout the day in pattern 1. The heat usage of pattern 2 reaches about 140 at about 10 am, but it sharply decreases to about 100 around 3 pm. After that, it increases significantly, and it peaks at about 145 between 8 and 11 pm. Pattern 2 correctly reflects the customer behaviors, which typically use heat when they are working during the day and at home during the evening.

The original dataset was partitioned into a training and testing set in order to analyze the daily heat usage pattern based on the identified heat usage patterns. Table 2 illustrates the data partitioning results.

**Table 2:** Daily heat load variation data partitioning results.

	# Pattern 1	# Pattern 2	Total
Whole dataset	29,563	13,517	43,080
Training set	23,650	10,813	34,463
Testing set	5913	2704	8617

#### Explaining the heat usage prediction models

The outcomes of the ML models can be visualized to explain how a model reaches a specific decision. This section performed shows various visualizations to explain the trained ML models.

**Figure 5:**
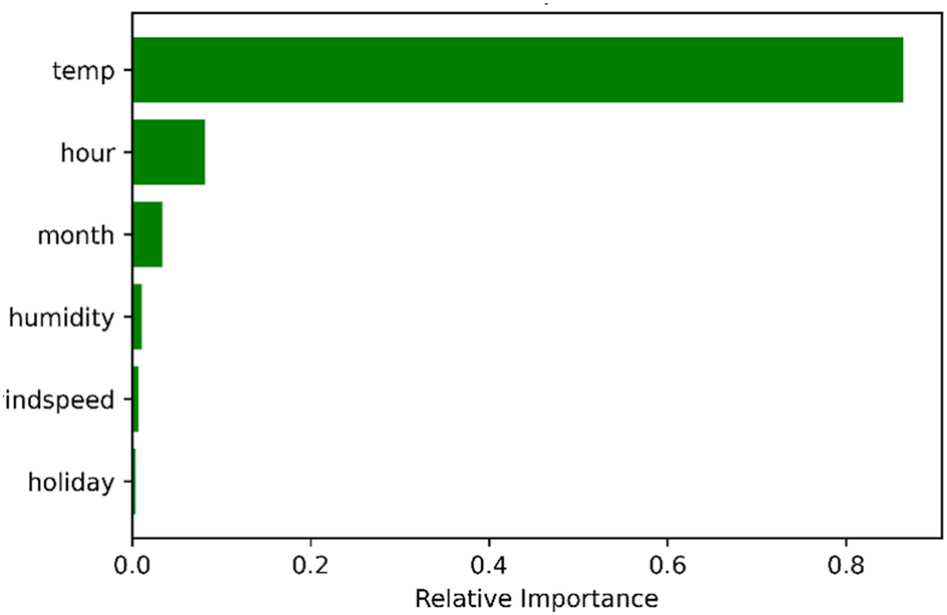
Feature importance using the mean decrease in impurity.

Firstly, the feature importance analysis technique was conducted, which assigned a score to each input feature based on its usefulness in predicting a target output. The features are described by their relative importance as a percentage of the most important feature. The relative importance is computed using the mean and the standard deviation of the collection of the impurity reduction with each weak learner. Figure 5 shows that the temperature and hour of the day are crucial to the model training process. The feature importance analysis correctly reflected the dataset because the customers’ heat usage pattern is largely affected by the outdoor temperature and the hour of the day.

The feature importance approach revealed that the hour feature significantly influenced the model's outcome, but it did not let researchers know how the feature influenced the model. Therefore, the partial dependence plot (PDP) method, which is a global and model agnostic XAI method, was implemented in the following experiments. PDP shows the marginal effect of a feature on the predictive value of the ML models^21^.

**Figure 6:**
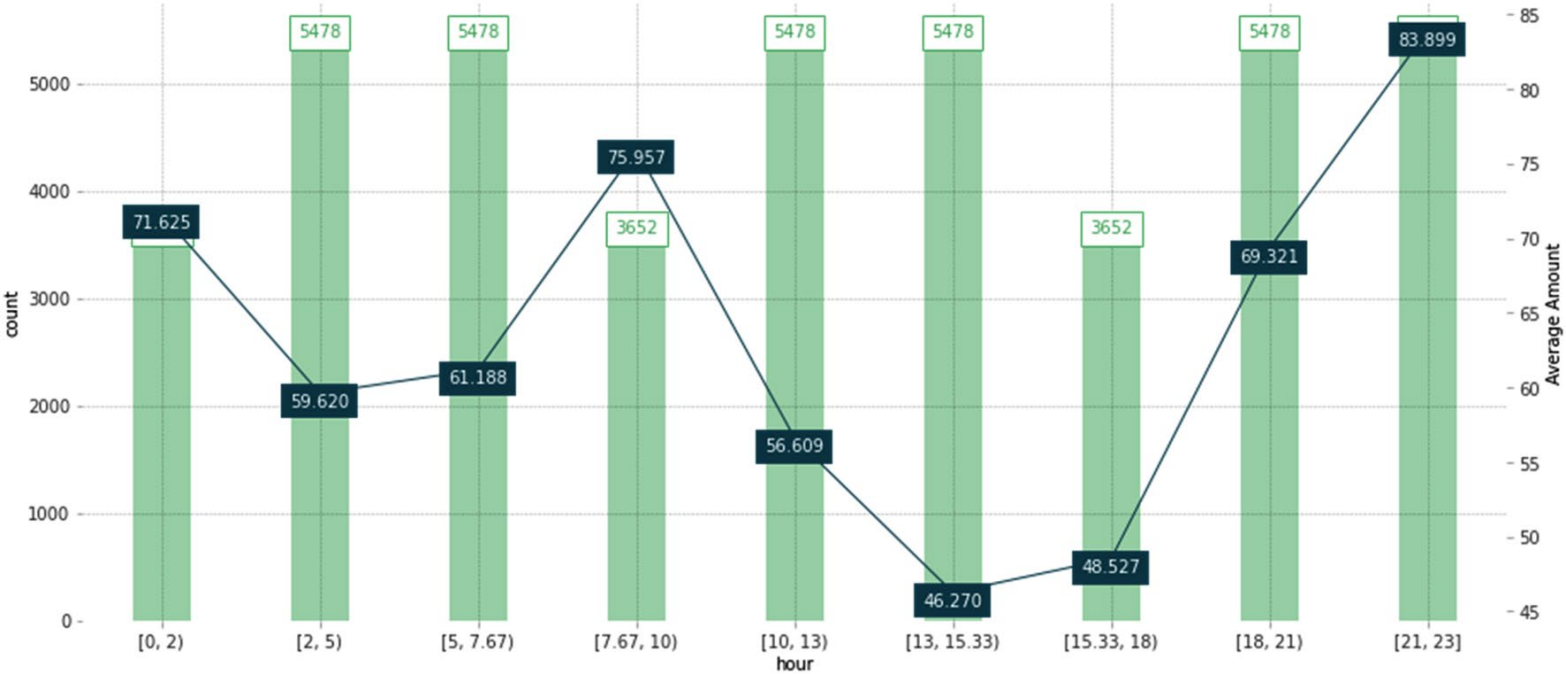
Target plot for the hour feature using the PDP.

Figure 6 illustrates the average target value using the PDP for the hour and temperature features. The graph is in line with the previously detected pattern 2 from “Dataset partition that is based on the daily heat usage variation” section, which shows that the heat usage peaks at two distinctive periods, which are 8–10 am in the morning and 9–11 pm in the evening. The heat usage sharply increases during these two periods of the day, because people stay indoors.

**Figure 7:**
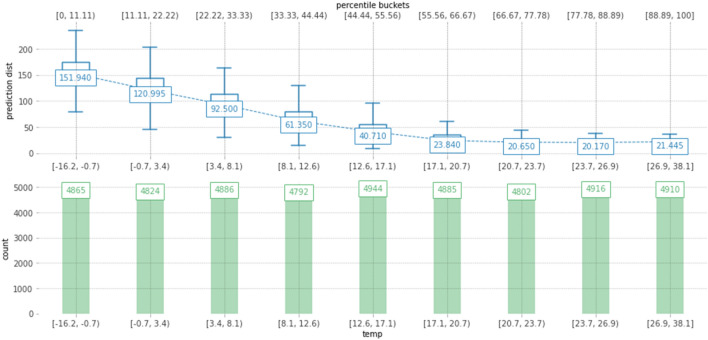
Actual heat usage prediction plot for the temperature feature using the PDP.

Figure 7 depicts the distribution of the actual heat usage prediction through different value ranges of the temperature feature. It is noticeable that the daily heat usage reaches the highest value, which is about 152 Gcal, when the outdoor temperature ranges from − 16.2° to − 0.7°, which is the typical temperature for the heating season during the winter. The daily heat usage value constantly decreases when the temperature range increases. The lowest daily heat usage, which is approximately 20 Gcal, is recorded when the temperature is between 23.7° and 26.9°.

#### Model performance analysis

Table 3 describes the heat usage prediction performance using three different ML models on the daily and seasonal heat load variations. In general, it can be seen that all the models performed well on the collected dataset. The XGBoost model achieved the highest ${{\varvec{R}}}^{2}$ of 0.88 for the seasonal heat usage scenario. The RMSE value is 10.4, and the MAE value is 11.7. On the other hand, the XGBoost and SVR showed better daily heat usage variation performances than the MLP. The ${{\varvec{R}}}^{2}$, RMSE, and MAE values are 0.9, 11.7, and 9, respectively for pattern 1, and they are 0.87, 12, and 9.23, respectively for pattern 2.

**Table 3:** Overall heat usage prediction performance using different ML algorithms on the validation dataset.

Heat usage variation	Algorithm	${R}^{2}$	RMSE	MAE
Daily (pattern 1)	SVR	**0.9**	**11.7**	**9**
	MLP	0.89	12.2	10.8
	XGBoost	**0.9**	**11.7**	**9**
Daily (pattern 2)	SVR	**0.87**	**12**	**9.23**
	MLP	0.8	15	11.7
	XGBoost	**0.87**	**12**	**9.23**
Seasonal	SVR	0.86	12.8	13.5
	MLP	0.84	13.7	14.7
	XGBoost	**0.88**	**10.4**	**11.7**

Significant values are in bold.

**Figure 8:**
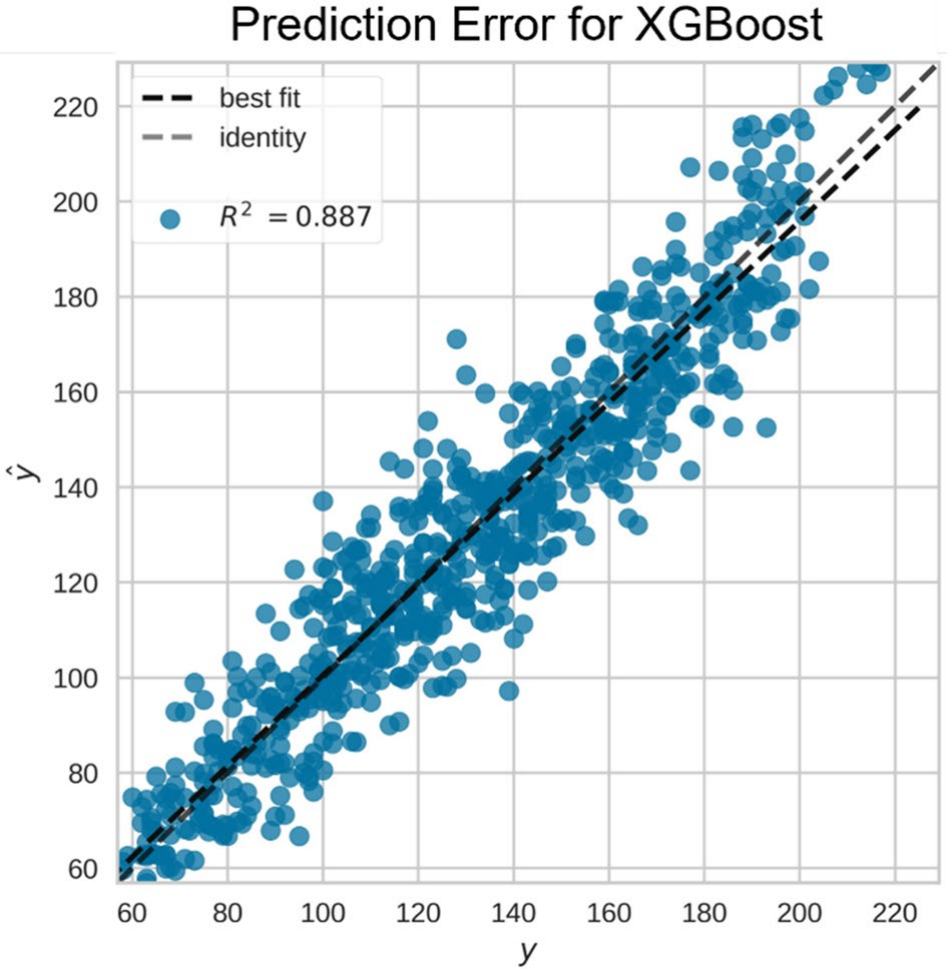
Prediction error plot on the seasonal heat variation test set using the XGBoost algorithm.

It can be witnessed from Table 3 that the XGBoost model shows the highest performance for both the seasonal and the daily heat variation scenarios. Figure 8 is the prediction error plot of the XGBoost algorithm on the seasonal variation, which plots the ground truth heat usage against the predicted values forecast by the XGBoost model. The best fit line is nearly fitted to the identity line with the ${R}^{2}$ value of 0.887, which shows a high correlation between the ground truth and the predicted target heat usage variable.

**Figure 9:**
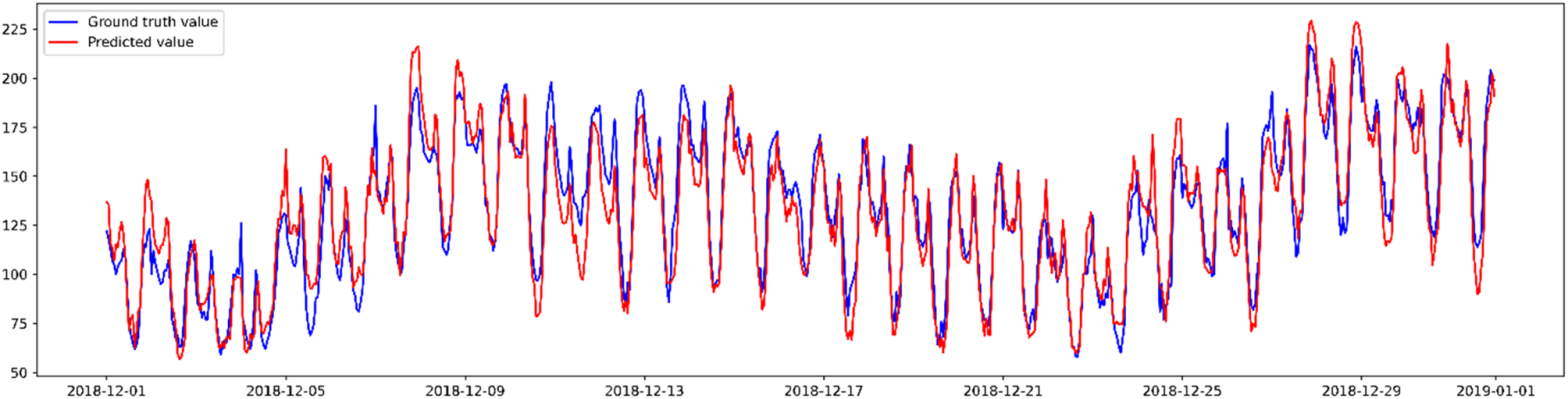
Daily heat load prediction results on the seasonal heat variation test set using the XGBoost algorithm.

Figure 9 shows a day-by-day comparison of the ground truth and the predicted heat usage on the testing set. Overall, the model predicted the heat usage appropriately with the predicted values, which were near the actual values. Also, the peak values and the bottom values were correctly predicted each day. However, the prediction accuracy decreased slightly due to the irregular customers' heat usage behavior on some specific days.

## Conclusion

Daily heat load prediction is necessary for DH companies to ensure an efficient heat supply and enable optimal heat transfer operations. This study introduces a daily heat usage prediction framework that uses standard ML algorithms, and it systematically analyzes the effect of the essential features on the models' outcomes.

In the suggested heat usage prediction framework, three additional pieces of information, including holidays, outdoor temperatures, wind speeds, and humidity, are chosen as the input features to train the prediction models in addition to the main historical hourly heat load information. Next, the data preprocessing was conducted on the raw dataset to reduce the misleading results. After that, the preprocessed data was fed into three well-known based learners, which included SVR, MLP, and XGBoost, to build the prediction models. The framework's performance was then evaluated using various evaluation metrics.

This study analyzes two main heat usage variations that include daily and seasonal, which is different from the previous studies. The XGBoost-based prediction model achieved the highest seasonal and daily heat load prediction accuracy. The recorded values of ${R}^{2}$, RMSE, and MAE of the seasonal variation are 0.88, 10.4, and 11.7, whereas they are 0.9, 11.7, and 9 for the daily variation. Finally, several explainable approaches, which included PDP and the feature importance, were implemented to give an in-depth view of the heat dataset. The visualization results correctly reflect that temperature and time are the most important features that significantly affect the model’s outcome.

In the future, more actions are required toward more advanced heat usage prediction strategies, such as multi-step ahead heat usage prediction tasks. Moreover, a bigger dataset and additional features can be added to increase the model's robustness.

## Data Availability

The datasets used and/or analysed during the current study available from the corresponding author on reasonable request.
