# Author Correction: Daily and seasonal heat usage patterns analysis in heat networks

**DOI:** 10.1038/s41598-022-15046-4

**Published:** 2022-06-23

**Authors:** L. Minh Dang, Sujin Lee, Yanfen Li, Chanmi Oh, Tan N. Nguyen, Hyoung-Kyu Song, Hyeonjoon Moon

**Affiliations:** 1 Department of Information and Communication Engineering, and Convergence Engineering for Intelligent Drone, Sejong University, Seoul, Republic of Korea; 2 Department of Artificial Intelligence, Sejong University, Seoul, Republic of Korea; 3 Department of Computer Science and Engineering, Sejong University, Seoul, Republic of Korea; 4 Department of Architectural Engineering, Sejong University, 209 Neungdong-ro, Gwangjin-gu, Seoul, 05006 Republic of Korea

Correction to: *Scientific Reports* https://doi.org/10.1038/s41598-022-13030-6, published online 02 June 2022

In the original version of this Article, Sujin Lee was incorrectly affiliated with ‘Department of Computer Science and Engineering, Sejong University, Seoul, Republic of Korea’. The correct affiliation is listed below.

Department of Artificial Intelligence, Sejong University, Seoul, Republic of Korea.

The original Article has been corrected.

